# Error rates of human reviewers during abstract screening in systematic reviews

**DOI:** 10.1371/journal.pone.0227742

**Published:** 2020-01-14

**Authors:** Zhen Wang, Tarek Nayfeh, Jennifer Tetzlaff, Peter O’Blenis, Mohammad Hassan Murad

**Affiliations:** 1 Evidence-based Practice Center, Mayo Clinic, Rochester, Minnesota, United States of America; 2 Robert D. and Patricia E. Kern Center for the Science of Health Care Delivery Mayo Clinic, Rochester, Minnesota, United States of America; 3 Evidence Partners, Ottawa, Ontario, Canada; Virginia Commonwealth University, UNITED STATES

## Abstract

**Background:**

Automated approaches to improve the efficiency of systematic reviews are greatly needed. When testing any of these approaches, the criterion standard of comparison (gold standard) is usually human reviewers. Yet, human reviewers make errors in inclusion and exclusion of references.

**Objectives:**

To determine citation false inclusion and false exclusion rates during abstract screening by pairs of independent reviewers. These rates can help in designing, testing and implementing automated approaches.

**Methods:**

We identified all systematic reviews conducted between 2010 and 2017 by an evidence-based practice center in the United States. Eligible reviews had to follow standard systematic review procedures with dual independent screening of abstracts and full texts, in which citation inclusion by one reviewer prompted automatic inclusion through the next level of screening. Disagreements between reviewers during full text screening were reconciled via consensus or arbitration by a third reviewer. A false inclusion or exclusion was defined as a decision made by a single reviewer that was inconsistent with the final included list of studies.

**Results:**

We analyzed a total of 139,467 citations that underwent 329,332 inclusion and exclusion decisions from 86 unique reviewers. The final systematic reviews included 5.48% of the potential references identified through bibliographic database search (95% confidence interval (CI): 2.38% to 8.58%). After abstract screening, the total error rate (false inclusion and false exclusion) was 10.76% (95% CI: 7.43% to 14.09%).

**Conclusions:**

This study suggests important false inclusion and exclusion rates by human reviewers. When deciding the validity of a future automated study selection algorithm, it is important to keep in mind that the gold standard is not perfect and that achieving error rates similar to humans may be adequate and can save resources and time.

## Introduction

Systematic review is a process to identify, select, synthesize and appraise all empirical evidence that fits pre-specified criteria to answer a specific research question. Since Archie Cochrane criticized lack of reliable evidence in medical care and called for “critical summary, by specialty or subspecialty, adapted periodically, of all relevant randomized control trials” in 1970s [1], systematic review has become the foundation of modern evidence based medicine. It is estimated that the annual publications of systematic reviews increased 2,728% from 1,024 in 1991 to 28,959 in 2014.[2]

Despite of the surging number of published systematic reviews in recent years, many systematic reviews employ suboptimal methodological approaches.[2–4] Rigorous systematic reviews require strict procedures with at least eight time-consuming steps.[5, 6] Significant time and resources are needed, with estimated 0.9 minutes, 7 minutes and 53 minutes spent per reference per reviewer on abstract screening, full text screening, and data extraction; respectively.[7, 8] One thousand potential studies retrieved from literature search required 952 hours to complete.[9]Therefore, methods to improve efficiency of systematic reviews without jeopardizing the validity are greatly needed.

In recent years, innovations have been proposed to accelerate the process of systematic reviews, including methods to simplify steps of systematic reviews (e.g., rapid systematic reviews)[10–13], and technology to facilitate literature retrieval, screening, and extraction.[7, 8, 14–21] Automation tools for systematic reviews, based on machine learning, text mining, and natural language processing, have particularly been popular with an estimated workload reduction from 30% to 70%.[14] Till July 2019, 39 tools have been completed and are available for “real-world” use.[22] However, innovations are not always perfect and may introduce additional "unintended" errors. A recent study found an automation tool used by health systems to identify patients with complex health needs led to significant racial bias.[23] Assessment of these automation tools for systematic reviews, thus, is critical for wide adoption in practice.[19, 24] No large scale test has been conducted. No conclusions have been made on whether and how to implement these automation tools. Theoretically, assessment of the automation tools can be treated as a classification problem: to determine whether a citation should be included or excluded. The standard outcome metrics are used, such as sensitivity, specificity, area under curve, positive predictive value. The standard of comparison (a.k.a., gold standard) is usually human reviewers. Yet, human reviewers make errors. There is lack of evidence of human errors in the process of systematic reviews.

Thus, we conducted this study to determine citation selection error rate (false inclusion and false exclusion rates) in systematic reviews conducted by pairs of independent human reviewers during abstract screening. These rates are currently unknown and can help in designing, testing and implementing automated approaches.

## Materials and methods

### Study design and data source

We searched all systematic reviews conducted by an evidence-based practice center in the United States. The evidence-based practice center is one of the 12 evidence-based practice centers designated and funded by U.S. Agency for Healthcare Research and Quality (AHRQ). It specializes in conducting systematic reviews and meta-analysis, and developing clinical practice guidelines, evidence dissemination and implementation tools, and related methodological research. Eligible systematic reviews had to 1) be started and finished between June 1, 2010 and Dec 31, 2017; 2) follow standard systematic review procedures [5]: 1) dual independent screening of abstracts and titles, abstract inclusion by one reviewer prompted automatic inclusion for full text screening; 2) dual independent screening of full text, disagreements between reviewers reconciled via consensus or arbitration by a third reviewer. The final included list of studies consisted of the studies after abstract screening, and full texting screening.; 3) use a web-based commercial systematic review software (DistillerSR, Evidence Partners Incorporated, Ottawa, Canada); and 4) be led by at least one of the core investigators of the evidence-based practice center. The investigation team consisted of a core group (10–15 investigators at any time period) and external collaborators with either methodological or content expertise. DistillerSR was used to facilitate abstracts and full texts screening and track all inclusion and exclusion decisions made by human reviewers. We did not use any automation algorithm in the included systematic reviews.

### Outcomes

**Fig 1:**
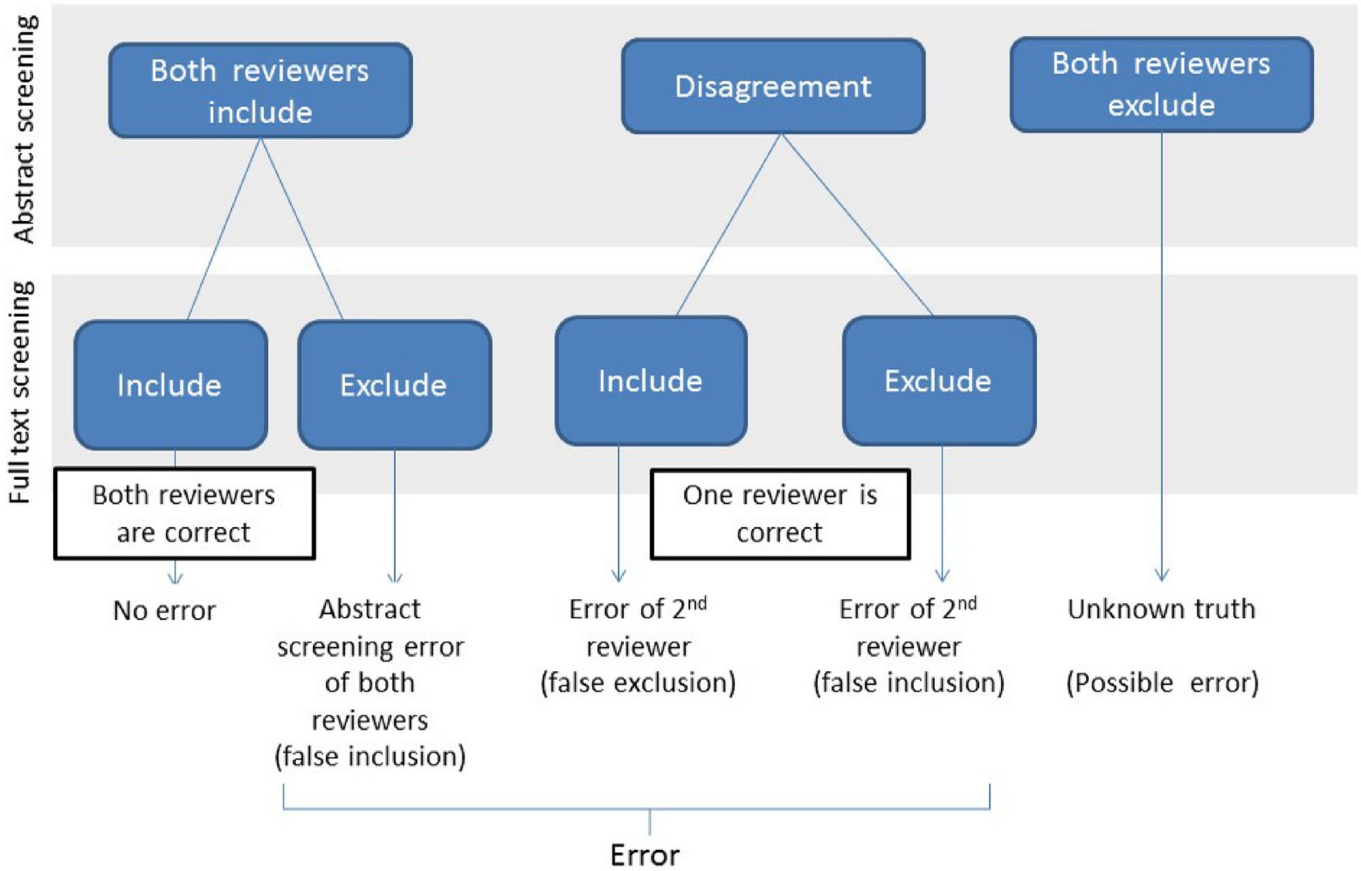
Errors occurred during systematic review abstract screening.

The main outcome of interest was error rate of human reviewers during abstract screening. An error was defined as a decision made by a single reviewer in abstract screening that was inconsistent (i.e., false inclusion or false exclusion) with the final included list of studies that that underwent abstract screening, and full texting screening and were eligible for data extraction and analysis (see Fig 1). We calculated error rate as the number of errors divided by the total number of screened abstracts (the total number of citations*2). We also estimated the overall abstract inclusion rate (defined as the number of eligible studies after abstract screening divided by the total number of citations), and the final inclusion rate (defined as the number of the final included studies divided by the total number of citations). In this study, we did not compare the performance between human reviewers and the automation algorithms integrated in DistillerSR.

### Statistical analysis

We calculated the outcomes of interest for each eligible systematic review. The mean of the outcomes across systematic reviews were the average outcomes of each study weighted by the inverse proportion to the variance of the denominator (total number of screened abstracts or total number of citations). The variance was estimated using the following formula:

$$V=\left\{\frac{n}{\left[sum\;w_{i}(n-1\right)]}\right\}sum\;w_{i}{\left(x_{i}-xbar\right)}^{2}$$

*xbar* = weight mean

All analyses were implemented with Stata version 15.1 (StataCorp LP, College Station, TX, USA).

## Results

A total of 25 systematic reviews were included in the analyses. These systematic reviews included 139,467 citations, representing 329,332 inclusion and exclusion decisions from 85 unique reviewers. Twenty-eight reviewers were core investigators from the evidence-based practice center; 57 were external collaborators with content or methodological expertise. Table 1 listed the characteristics of the included systematic reviews.

**Table 1:** Characteristics of the included systematic reviews.

Characteristics	Results
Systematic reviews	25
Time period	June 2010 to December 2017
Citations from literature search	139,467
Inclusion and exclusion decisions	329,332
Decisions after abstract screening	278,934
Decisions after full text screening	50,398
Systematic reviewers	85
From the core team	28
External content or methodological experts	57
Clinical area	
Cardiovascular medicine	1
Mental health	2
Primary care	3
Pulmonology and critical care	2
Cardiovascular medicine	1
Endocrinology	7
Hematology	2
Health care delivery research	4
Urology	3
Review question type	
Methodology	4
Diagnostic/Screening/Prognostic	4
Treatment	17

Abstract screening inclusion rate was 18.07% (95% CI: 12.65% to 23.48%) of the citations identified through literature search. Final inclusion rate after full text screening was 5.48% of the citations identified through literature search included in the systematic review (95% confidence interval (CI): 2.38% to 8.58%). The total error rate was 10.76% (95% CI: 7.43% to 14.09%). The error rates and inclusion rates varied by clinical area and type of review questions (Table 2).

**Table 2:** Error and inclusion rates by topic area and type of review questions.

	Final inclusion rate (95% CI) dual process	Abstract inclusion rate (95% CI) dual process	Error rate (95% CI)
**Overall (n = 25)**	5.48% (2.38% to 8.58%)	18.07% (12.65% to 23.48%)	10.76% (7.43% to 14.09%)
**Clinical Area**			
**Cardiovascular medicine (n = 1)**	1.59%	23.92%	17.73%
**Mental health (n = 2)**	2.10% (0% to 20.15%)	9.92% (0.77% to19.06%)	6.43% (0% to 18.19%)
**Primary care (n = 3)**	5.39% (0% to 13.19%)	28.00% (20.85%, 35.15%)	21.11% (5.15% to 37.08%)
**Pulmonology and critical care (n = 2)**	1.12% (0% to 2.43%)	9.13% (05 to 38.18%)	6.68% (0% to 42.04%)
**Cardiovascular medicine (n = 1)**	1.93%	18.56%	19.16%
**Endocrinology (n = 7)**	5.91% (2.89% to 8.94%)	20.40% (9.70% to 31.09%)	12.23% (4.76% to 19.70%)
**Hematology (n = 2)**	6.69% (0% to 37.32%)	11.00% (0% to 40.13%)	5.76% (0% to 24.27%)
**Health care delivery research (n = 4)**	2.18% (0% to 6.86%)	14.55% (0% to 43.35%)	8.73% (0% to 29.03%)
**Urology (n = 3)**	23.77% (0% to 57.85%)	42.85% (24.92% to 60.79%)	17.17% (0% to 36.07%)
**Review question type**			
**Methodology**	2.18% (0.00% to 6.86%)	14.55% (0% to 43.35%)	8.73% (0.00% to 29.03%
**Diagnostic/Screening/Prognostic**	7.83% (3.56% to 12.11%)	25.99% (0% to 57.82%)	14.97% (0% to 34.38%)
**Treatment**	5.86% (1.47% to 10.26%)	17.64% (12.00% to 23.29%)	10.57% (7.32% to 13.83%)

## Discussion

In this cohort of 25 systematic reviews, covering 9 clinical areas and 3 types of clinical questions, a total of 329,332 screening decisions (inclusion vs. exclusion) were made by 85 human reviewers. The error rate (false inclusion and false exclusion) during abstract screening was 10.76%, which varied from 5.76% to 21.11%, depending on clinical areas and question types.

### Implications

A rigorous systematic review follows strict approaches and requires significant resource and time to complete, which typically lasts 6–18 months by a team of human reviewers.[25] Automation tools have the potential to mimic human activities in systematic review tasks and gained popularity in academia and industry. However, validity of the automation tools has yet to be established. [19, 24] It is intuitive to assume that these tools should achieve a zero error rate in order to be implemented to generate evidence used for decision-making (i.e., 100% sensitivity and 100% specificity).

Human reviewers have been used as the “gold standard” in evaluating the automation tools. However, similar to those “gold standards” used in clinical medicine, 100% accuracy is unlikely in reality. We found 10.76% error rate made by human reviewers in abstract screening (an error about 1 in 9 abstracts). This error rate also varied from topics and types of questions. Thus, when developing and refining an automation tool, achieving error rates similar to humans may be adequate. If this is the case, then these tools can serve as a single reviewer that gets paired with a second human reviewer.

### Limitations

The sample size is relatively small, especially as we further stratify by clinical areas. The findings may not be generalizable to other systematic review questions or topics. The human reviewers who conducted these systematic reviews had a wide range of content knowledge and methodological experience (from minimum 1 year to over 10 years), which can be quite different from other review teams. In our practice, citations from abstract screening were automatically included when conflicts between two independent reviewers emerged. The abstract inclusion rate and final inclusion rate resulting from this approach can be higher than those of the teams who resolve conflicts in abstract screening. When both reviewers agree on excluding an abstract, this abstract disappears from the process; thus, a dual erroneous exclusion cannot be assessed. We were not able to evaluate error rate during full text screening as we did not track the conflicts between reviewers. Lastly, while we call judgments in study selection that are inconsistent with the final inclusion as errors, we acknowledge that these errors could be due to poor reporting and insufficient data provided in the published abstract. Thus, they may not be avoidable and they are not the fault of human reviewers. In summary, this study is an initial step to evaluate human errors in systematic reviews. Future studies need to evaluate different systematic review approaches (e.g., rapid systematic review, scoping review), clinical areas, and review questions. It is also important to increase the number of systematic reviews involved in the evaluation and include other EPC or non-EPC institutions.

## Conclusions

This study of 329,332 abstract screening decisions made by a large, diverse group of systematic reviewers suggests important false inclusion and exclusion rates by human reviewers. When deciding the validity of a future automated study selection algorithm, it is important to keep in mind that the gold standard is not perfect and that achieving error rates similar to humans is likely adequate and can save resources and time.

## Supporting information

S1 Appendix (DOCX)

## References

[1] Cochrane AL. 1931–1971: a critical review, with particular reference to the medical profession. Medicines for the year. 2000;1979:1.

[2] Ioannidis JP. The Mass Production of Redundant, Misleading, and Conflicted Systematic Reviews and Meta-analyses. Milbank Q. 2016;94(3):485–514. Epub 2016/09/14. 10.1111/1468-0009.12210 27620683 PMC5020151

[3] Page MJ, Altman DG, McKenzie JE, Shamseer L, Ahmadzai N, Wolfe D, et al Flaws in the application and interpretation of statistical analyses in systematic reviews of therapeutic interventions were common: a cross-sectional analysis. J Clin Epidemiol. 2018;95:7–18. Epub 2017/12/06. 10.1016/j.jclinepi.2017.11.022 . 29203419

[4] Baudard M, Yavchitz A, Ravaud P, Perrodeau E, Boutron I. Impact of searching clinical trial registries in systematic reviews of pharmaceutical treatments: methodological systematic review and reanalysis of meta-analyses. BMJ. 2017;356:j448 Epub 2017/02/19. 10.1136/bmj.j448 28213479 PMC5421496

[5] Higgins JP, Green S. Cochrane handbook for systematic reviews of interventions: John Wiley & Sons; 2011.

[6] Murad MH, Montori VM, Ioannidis JP, Jaeschke R, Devereaux P, Prasad K, et al How to read a systematic review and meta-analysis and apply the results to patient care: users’ guides to the medical literature. Jama. 2014;312(2):171–9. 10.1001/jama.2014.5559 25005654

[7] Wang Z, Asi N, Elraiyah TA, Abu Dabrh AM, Undavalli C, Glasziou P, et al Dual computer monitors to increase efficiency of conducting systematic reviews. J Clin Epidemiol. 2014;67(12):1353–7. Epub 2014/08/03. 10.1016/j.jclinepi.2014.06.011 . 25085736

[8] Wang Z, Noor A, Elraiyah T, Murad M, editors. Dual monitors to increase efficiency of conducting systematic reviews. 21st Cochrane Colloquium; 2013. 10.1016/j.jclinepi.2014.06.011 25085736

[9] Allen IE, Olkin I. Estimating time to conduct a meta-analysis from number of citations retrieved. JAMA. 1999;282(7):634–5. Epub 1999/10/12. 10.1001/jama.282.7.634 . 10517715

[10] Khangura S, Konnyu K, Cushman R, Grimshaw J, Moher D. Evidence summaries: the evolution of a rapid review approach. Syst Rev. 2012;1:10 Epub 2012/05/17. 10.1186/2046-4053-1-10 22587960 PMC3351736

[11] Hailey D, Corabian P, Harstall C, Schneider W. The use and impact of rapid health technology assessments. International journal of technology assessment in health care. 2000;16(2):651–6. 10.1017/s0266462300101205 10932429

[12] Patnode CD, Eder ML, Walsh ES, Viswanathan M, Lin JS. The use of rapid review methods for the US Preventive Services Task Force. American journal of preventive medicine. 2018;54(1):S19–S25. 29254522 10.1016/j.amepre.2017.07.024

[13] Ganann R, Ciliska D, Thomas H. Expediting systematic reviews: methods and implications of rapid reviews. Implement Sci. 2010;5:56 Epub 2010/07/21. 10.1186/1748-5908-5-56 20642853 PMC2914085

[14] O’Mara-Eves A, Thomas J, McNaught J, Miwa M, Ananiadou S. Using text mining for study identification in systematic reviews: a systematic review of current approaches. Systematic reviews. 2015;4(1):5. 25588314 10.1186/2046-4053-4-5 PMC4320539

[15] Li D, Wang Z, Wang L, Sohn S, Shen F, Murad MH, et al A Text-Mining Framework for Supporting Systematic Reviews. Am J Inf Manag. 2016;1(1):1–9. Epub 2017/10/27. 29071308 PMC5653323

[16] Li D, Wang Z, Shen F, Murad MH, Liu H, editors. Towards a multi-level framework for supporting systematic review—A pilot study. 2014 IEEE International Conference on Bioinformatics and Biomedicine (BIBM); 2014: IEEE.

[17] Alsawas M, Alahdab F, Asi N, Li DC, Wang Z, Murad MH. Natural language processing: use in EBM and a guide for appraisal. BMJ Evidence-Based Medicine. 2016;21(4):136–8. 10.1136/ebmed-2016-110437 27284128

[18] Cohen AM, Hersh WR, Peterson K, Yen P-Y. Reducing workload in systematic review preparation using automated citation classification. Journal of the American Medical Informatics Association. 2006;13(2):206–19. 10.1197/jamia.M1929 16357352 PMC1447545

[19] Beller E, Clark J, Tsafnat G, Adams C, Diehl H, Lund H, et al Making progress with the automation of systematic reviews: principles of the International Collaboration for the Automation of Systematic Reviews (ICASR). Syst Rev. 2018;7(1):77 Epub 2018/05/21. 10.1186/s13643-018-0740-7 29778096 PMC5960503

[20] O’Connor AM, Tsafnat G, Gilbert SB, Thayer KA, Shemilt I, Thomas J, et al Still moving toward automation of the systematic review process: a summary of discussions at the third meeting of the International Collaboration for Automation of Systematic Reviews (ICASR). Syst Rev. 2019;8(1):57 Epub 2019/02/23. 10.1186/s13643-019-0975-y 30786933 PMC6381675

[21] Bannach-Brown A, Przybyla P, Thomas J, Rice ASC, Ananiadou S, Liao J, et al Machine learning algorithms for systematic review: reducing workload in a preclinical review of animal studies and reducing human screening error. Syst Rev. 2019;8(1):23 Epub 2019/01/17. 10.1186/s13643-019-0942-7 30646959 PMC6334440

[22] SR Toolbox 2019 [cited 2019 August 6]. http://systematicreviewtools.com/index.php.

[23] Obermeyer Z, Powers B, Vogeli C, Mullainathan S. Dissecting racial bias in an algorithm used to manage the health of populations. Science. 2019;366(6464):447–53. 10.1126/science.aax2342 31649194

[24] O’Connor AM, Tsafnat G, Thomas J, Glasziou P, Gilbert SB, Hutton B. A question of trust: can we build an evidence base to gain trust in systematic review automation technologies? Systematic Reviews. 2019;8(1):143 10.1186/s13643-019-1062-0 31215463 PMC6582554

[25] Smith V, Devane D, Begley CM, Clarke M. Methodology in conducting a systematic review of systematic reviews of healthcare interventions. BMC Med Res Methodol. 2011;11(1):15 Epub 2011/02/05. 10.1186/1471-2288-11-15 21291558 PMC3039637

