# Supplementary material for: Error rates of human reviewers during abstract screening in systematic reviews
Source: PLoS One. 2020 Jan 14;15(1):e0227742. doi: 10.1371/journal.pone.0227742 (PMC6959565; doi:10.1371/journal.pone.0227742)
Supplement: S1 Appendix — (DOCX) [file pone.0227742.s001.docx]

**PLoS One Supporting Information Appendix S1
Article title: Error Rates of Human Reviewers During Abstract Screening in Systematic Reviews**

Author: Zhen Wang, Tarek Nayfeh, Jennifer Tetzlaff, Peter O’Blenis, Mohammad Hassan Murad

S1 Table A. List of the included systematic reviews

**Table A. List of the included systematic reviews**

| Number | The included systematic reviews |
| --- | --- |
| 1 | Comparative Effectiveness and Safety of Cognitive Behavioral Therapy and Pharmacotherapy for Childhood Anxiety Disorders: A Systematic Review and Meta-analysis(1) |
| 2 | Glucocorticoid Replacement Regimens in Chronic Adrenal Insufficiency: A Systematic Review and Meta-Analysis(2) |
| 3 | Management of Anaplastic Thyroid Cancer: A Systematic Review (publication pending) |
| 4 | Adolescent Perspectives on Medicine-taking in Asthma – Systematic Review and Thematic Synthesis of Qualitative Studies (publication pending) |
| 5 | Castration-resistant Prostate Cancer: AUA Guideline(3) |
| 6 | Early Detection of Prostate Cancer: AUA Guideline(4) |
| 7 | A Systematic Review of Patient-Reported Measures of Burden of Treatment in Three Chronic Diseases(5) |
| 8 | A Systematic Review and Meta-Analysis of Revascularization Outcomes of Infrainguinal Chronic Limb-threatening Ischemia(6) |
| 9 | Non-pharmacological Treatment of Depression: A Systematic Review and Evidence Map(7) |
| 10 | Impact of Decision Aids Used During Clinical Encounters on Clinician Outcomes and Consultation Length: A Systematic Review(8) |
| 11 | Behavioral Interventions for Chronic Disease: A Systematic Review (publication pending) |
| 12 | Readability of Online Health Information: A Meta-Narrative Systematic Review(9) |
| 13 | Hydroxyurea for Sickle Cell Disease: A Systematic Review of Benefits, Harms and Barriers of Utilization(10) |
| 14 | Surgical Management of Kidney Stones: A Systematic Review and Meta-Analysis (publication pending) |
| 15 | Outcomes of Parathyroidectomy in Patients with Primary Hyperparathyroidism: A Systematic Review and Meta-analysis(11) |
| 16 | Do We Need New Quality Indicators in the PICU? A Systematic Review and Meta-Analysis (publication pending) |
| 17 | The Management of Sickle Cell Disease Complications: A Systematic Review(10) |
| 18 | A Systematic Review of Shared Decision Making Interventions in Chronic Conditions (publication pending) |
| 19 | The Cortisol Stress Response Induced by Surgery: A Systematic Review and Meta-Analysis(12) |
| 20 | Testosterone Therapy and Venous Thromboembolism: A Systematic Review and Meta-Analysis(13) |
| 21 | Too much medicine? A Systematic Review and Meta-Analysis (publication pending) |
| 22 | Effectiveness of Surgical Interventions for Thoracic Aortic Aneurysms: A Systematic Review and Meta-Analysis(14) |
| 23 | The Accuracy of Thyroid Nodule Ultrasound to Predict Thyroid Cancer: A Systematic Review and Meta-Analysis(15) |
| 24 | Treatment of Pediatric Obesity: An Umbrella Systematic Review(16) |
| 25 | Drugs Commonly Associated with Weight Change: A Systematic Review and Meta-Analysis(17) |

Table B. Supporting data used in the analyses

| Publication | Clinial Area | Review Question Type | Total Refs | After Abstract Screening | Final Included in Systematic Review | Abstract Screening Decisions | Full Text Screening Decisions | Consensus False Inclusion after Abstract Screening | False Inclusion after Abstract Screening (including consensus false inclusion) | False Exclusion after Abstract Screening | Total Errors |
| --- | --- | --- | --- | --- | --- | --- | --- | --- | --- | --- | --- |
| Glucocorticoid Replacement Regimens in Chronic Adrenal Insufficiency: A Systematic Review and Meta-Analysis | endocrinology | treatment | 2726 | 252 | 25 | 5452 | 504 | 227 | 241 | 10 | 251 |
| Management of Anaplastic Thyroid Cancer: A Systematic Review | endocrinology | treatment | 2834 | 819 | 320 | 5668 | 1638 | 499 | 1328 | 15 | 1343 |
| Comparative Effectiveness and Safety of Cognitive Behavioral Therapy and Pharmacotherapy for Childhood Anxiety Disorders: A Systematic Review and Meta-analysis | mental health | treatment | 32156 | 3289 | 479 | 64312 | 6578 | 2810 | 4334 | 57 | 4391 |
| Adolescent Perspectives on Medicine-taking in Asthma – Systematic Review and Thematic Synthesis of Qualitative Studies | pulmonology and critical care | treatment | 4937 | 526 | 52 | 9874 | 1052 | 474 | 829 | 14 | 843 |
| Castration-resistant Prostate Cancer: AUA Guideline | urology | treatment | 2728 | 859 | 297 | 5456 | 1718 | 562 | 1133 | 31 | 1164 |
| Early Detection of Prostate Cancer: AUA Guideline | urology | diagnostic/screening/prognostic | 2972 | 1393 | 337 | 5944 | 2786 | 1056 | 1511 | 53 | 1564 |
| A Systematic Review of Patient-Reported Measures of Burden of Treatment in Three Chronic Diseases | health care delivery research | methodology | 5708 | 974 | 118 | 11416 | 1948 | 856 | 984 | 34 | 1018 |
| A Systematic Review and Meta-Analysis of Revascularization Outcomes of Infrainguinal Chronic Limb-threatening Ischemia | cardiovascular medicine | treatment | 3163 | 587 | 61 | 6326 | 1174 | 526 | 1200 | 12 | 1212 |
| Non-pharmacological Treatment of Depression: A Systematic Review and Evidence Map | mental health | treatment | 5988 | 494 | 323 | 11976 | 988 | 171 | 347 | 166 | 513 |
| Behavioral Interventions for Chronic Disease: A Systematic Review | health care delivery research | methodology | 6694 | 408 | 30 | 13388 | 816 | 378 | 275 | 15 | 290 |
| Ghost Protocol the Resurrection | primary care | treatment | 5070 | 1305 | 164 | 10140 | 2610 | 1141 | 1559 | 88 | 1647 |
| Readability of Online Health Information: A Meta-Narrative Systematic Review | health care delivery research | methodology | 3625 | 1757 | 286 | 7250 | 3514 | 1471 | 2405 | 13 | 2418 |
| Hydroxyurea for Sickle Cell Disease: A Systematic Review of Benefits, Harms and Barriers of Utilization | hematology | treatment | 2604 | 193 | 76 | 5208 | 386 | 117 | 158 | 23 | 181 |
| Surgical Management of Kidney Stones: A Systematic Review and Meta-Analysis | urology | treatment | 7260 | 3302 | 2446 | 14520 | 6604 | 856 | 1131 | 592 | 1723 |
| Outcomes of Parathyroidectomy in Patients with Primary Hyperparathyroidism: A Systematic Review and Meta-analysis | endocrinology | treatment | 3141 | 792 | 291 | 6282 | 1584 | 501 | 623 | 202 | 825 |
| Do We Need New Quality Indicators in the PICU? A Systematic Review and Meta-Analysis | pulmonology and critical care | treatment | 2193 | 125 | 28 | 4386 | 250 | 97 | 102 | 8 | 110 |
| The Management of Sickle Cell Disease Complications: A Systematic Review | hematology | treatment | 6378 | 795 | 525 | 12756 | 1590 | 270 | 390 | 463 | 853 |
| Impact of Decision Aids Used During Clinical Encounters on Clinician Outcomes and Consultation Length: A Systematic Review | health care delivery research | methodology | 6888 | 196 | 66 | 13776 | 392 | 130 | 260 | 16 | 276 |
| The Cortisol Stress Response Induced by Surgery: A Systematic Review and Meta-Analysis | endocrinology | diagnostic/screening/prognostic | 7619 | 650 | 439 | 15238 | 1300 | 211 | 520 | 121 | 641 |
| Testosterone Therapy and Venous Thromboembolism: A Systematic Review and Meta-Analysis | endocrinology | treatment | 11225 | 2117 | 405 | 22450 | 4234 | 1712 | 2206 | 231 | 2437 |
| Too much medicine? A Systematic Review and Meta-Analysis | endocrinology | diagnostic/screening/prognostic | 3356 | 1429 | 331 | 6712 | 2858 | 1098 | 1499 | 95 | 1594 |
| Effectiveness of Surgical Interventions for Thoracic Aortic Aneurysms: A Systematic Review and Meta-Analysis | cardiovascular medicine | treatment | 3763 | 900 | 60 | 7526 | 1800 | 840 | 1273 | 61 | 1334 |
| Sonographic Diagnosis of Thyroid Cancer | endocrinology | diagnostic/screening/prognostic | 1646 | 580 | 114 | 3292 | 1160 | 466 | 868 | 2 | 870 |
| Treatment of Pediatric Obesity: An Umbrella Systematic Review | primary care | treatment | 1245 | 388 | 57 | 2490 | 776 | 331 | 744 | 4 | 748 |
| Drugs Commonly Associated with Weight Change: A Systematic Review and Meta-Analysis | primary care | treatment | 3548 | 1069 | 311 | 7096 | 2138 | 758 | 1706 | 64 | 1770 |

**Reference**

1. Wang Z, Whiteside SPH, Sim L, Farah W, Morrow AS, Alsawas M, et al. Comparative Effectiveness and Safety of Cognitive Behavioral Therapy and Pharmacotherapy for Childhood Anxiety Disorders: A Systematic Review and Meta-analysis. JAMA pediatrics. 2017;171(11):1049-56.

2. Al Nofal A, Bancos I, Benkhadra K, Ospina NM, Javed A, Kapoor E, et al. Glucocorticoid Replacement Regimens in Chronic Adrenal Insufficiency: A Systematic Review and Meta-Analysis. Endocrine practice : official journal of the American College of Endocrinology and the American Association of Clinical Endocrinologists. 2017;23(1):17-31.

3. Cookson MS, Roth BJ, Dahm P, Engstrom C, Freedland SJ, Hussain M, et al. Castration-resistant prostate cancer: AUA Guideline. The Journal of urology. 2013;190(2):429-38.

4. Carter HB, Albertsen PC, Barry MJ, Etzioni R, Freedland SJ, Greene KL, et al. Early detection of prostate cancer: AUA Guideline. The Journal of urology. 2013;190(2):419-26.

5. Eton DT, Elraiyah TA, Yost KJ, Ridgeway JL, Johnson A, Egginton JS, et al. A systematic review of patient-reported measures of burden of treatment in three chronic diseases. Patient related outcome measures. 2013;4:7-20.

6. Almasri J, Adusumalli J, Asi N, Lakis S, Alsawas M, Prokop LJ, et al. A systematic review and meta-analysis of revascularization outcomes of infrainguinal chronic limb-threatening ischemia. Journal of vascular surgery. 2018;68(2):624-33.

7. Farah WH, Alsawas M, Mainou M, Alahdab F, Farah MH, Ahmed AT, et al. Non-pharmacological treatment of depression: a systematic review and evidence map. Evidence-based medicine. 2016;21(6):214-21.

8. Dobler CC, Sanchez M, Gionfriddo MR, Alvarez-Villalobos NA, Singh Ospina N, Spencer-Bonilla G, et al. Impact of decision aids used during clinical encounters on clinician outcomes and consultation length: a systematic review. BMJ quality & safety. 2019;28(6):499-510.

9. Daraz L, Morrow AS, Ponce OJ, Farah W, Katabi A, Majzoub A, et al. Readability of Online Health Information: A Meta-Narrative Systematic Review. American journal of medical quality : the official journal of the American College of Medical Quality. 2018;33(5):487-92.

10. National Heart L, Institute B. Evidence-based management of sickle cell disease. 2014. 2014.

11. Singh Ospina NM, Rodriguez-Gutierrez R, Maraka S, Espinosa de Ycaza AE, Jasim S, Castaneda-Guarderas A, et al. Outcomes of Parathyroidectomy in Patients with Primary Hyperparathyroidism: A Systematic Review and Meta-analysis. World J Surg. 2016;40(10):2359-77.

12. Prete A, Yan Q, Al-Tarrah K, Akturk HK, Prokop LJ, Alahdab F, et al. The cortisol stress response induced by surgery: A systematic review and meta-analysis. Clinical endocrinology. 2018;89(5):554-67.

13. Houghton DE, Alsawas M, Barrioneuvo P, Tello M, Farah W, Beuschel B, et al. Testosterone therapy and venous thromboembolism: A systematic review and meta-analysis. Thrombosis research. 2018;172:94-103.

14. Alsawas M, Zaiem F, Larrea-Mantilla L, Almasri J, Erwin PJ, Upchurch GR, Jr., et al. Effectiveness of surgical interventions for thoracic aortic aneurysms: A systematic review and meta-analysis. Journal of vascular surgery. 2017;66(4):1258-68.e8.

15. Brito JP, Gionfriddo MR, Al Nofal A, Boehmer KR, Leppin AL, Reading C, et al. The accuracy of thyroid nodule ultrasound to predict thyroid cancer: systematic review and meta-analysis. The Journal of clinical endocrinology and metabolism. 2014;99(4):1253-63.

16. Rajjo T, Mohammed K, Alsawas M, Ahmed AT, Farah W, Asi N, et al. Treatment of Pediatric Obesity: An Umbrella Systematic Review. The Journal of clinical endocrinology and metabolism. 2017;102(3):763-75.

17. Domecq JP, Prutsky G, Leppin A, Sonbol MB, Altayar O, Undavalli C, et al. Clinical review: Drugs commonly associated with weight change: a systematic review and meta-analysis. The Journal of clinical endocrinology and metabolism. 2015;100(2):363-70.
