# Supplementary material for: Error rates of human reviewers during abstract screening in systematic reviews
Source: PLoS One. 2020 Jan 14;15(1):e0227742. doi: 10.1371/journal.pone.0227742 (PMC6959565; doi:10.1371/journal.pone.0227742)
Supplement: Attachment — Submitted filename: PONE-D-19-26633_reviewer.pdf [file pone.0227742.s002.pdf]

PLOS ONE

Error rates of human reviewers during abstract screening in systematic reviews

--Manuscript Draft--

**Manuscript Number:**	PONE-D-19-26633
**Article Type:**	Research Article
**Full Title:**	Error rates of human reviewers during abstract screening in systematic reviews
**Short Title:**	Error rates of human reviewers in systematic reviews
**Corresponding Author:**	Zhen Wang<br>Mayo Clinic<br>Rochester, UNITED STATES
**Keywords:**	systematic review automation, human error
**Abstract:**	Background<br>Automated approaches to improve the efficiency of systematic reviews are greatly needed. When testing any of these approaches, the criterion standard of comparison (gold standard) is usually human reviewers. Yet, human reviewers make errors in inclusion and exclusion of references.<br>Objectives<br>To determine citation false inclusion and false exclusion rates during abstract screening by pairs of independent reviewers. These rates can help in designing, testing and implementing automated approaches.<br>Methods<br>We identified all systematic reviews conducted between 2010 and 2017 by an evidence-based practice center in the United States. Eligible reviews had to follow standard systematic review procedures with dual independent screening of abstracts and full texts in which citation inclusion by one reviewer prompted automatic inclusion through the next level of screening. Disagreements between reviewers were reconciled via consensus or arbitration by a third reviewer. A false inclusion or exclusion was defined as a decision made by a single reviewer that was inconsistent with the final included list of studies that underwent full text screening and consensus, data extraction and analysis.<br>Results<br>We analyzed a total of 139,467 citations that underwent 329,332 inclusion and exclusion decisions from 86 unique reviewers. The final systematic reviews included 5.48% of the potential references identified through bibliographic database search (95% confidence interval (CI): 2.38% to 8.58%). After abstract screening, the total error rate (false inclusion and false exclusion) was 10.76% (95% CI: 7.43% to 14.09%).<br>Conclusions<br>This study suggests important false inclusion and exclusion rates. When deciding about the validity of a future automated study selection algorithm, it is important to keep in mind that the gold standard is not perfect and that achieving error rates similar to humans is likely adequate and can save resources and time. A dual process of an automated tool and a human reviewer is recommended.
**Order of Authors:**	Zhen Wang
	Tarek Nayfeh
	Jennifer Tetzlaff
	Peter O’Blenis
	Mohammad Hassan Murad
**Additional Information:**	
**Question**	**Response**
**Financial Disclosure**<br><br>Enter a financial disclosure statement that	The author(s) received no specific funding for this work.

Powered by Editorial Manager® and ProduXion Manager® from Aries Systems Corporation

describes the sources of funding for the work included in this submission. Review the submission guidelines for detailed requirements. View published research articles from *PLOS ONE* for specific examples.<br><br>This statement is required for submission and **will appear in the published article** if the submission is accepted. Please make sure it is accurate.<br><br>**Unfunded studies**<br>Enter: *The author(s) received no specific funding for this work.*<br><br>**Funded studies**<br>Enter a statement with the following details:<br>• Initials of the authors who received each award<br>• Grant numbers awarded to each author<br>• The full name of each funder<br>• URL of each funder website<br>• Did the sponsors or funders play any role in the study design, data collection and analysis, decision to publish, or preparation of the manuscript?<br>• **NO** - Include this sentence at the end of your statement: *The funders had no role in study design, data collection and analysis, decision to publish, or preparation of the manuscript.*<br>• **YES** - Specify the role(s) played.<br><br>* typeset	
**Competing Interests**<br><br>Use the instructions below to enter a competing interest statement for this submission. On behalf of all authors, disclose any competing interests that could be perceived to bias this work—acknowledging all financial support and any other relevant financial or non-financial competing interests.<br><br>This statement **will appear in the published article** if the submission is accepted. Please make sure it is accurate. View published research articles from *PLOS ONE* for specific examples.	The authors have declared that no competing interests exist.

**NO authors have competing interests**<br><br>Enter: *The authors have declared that no competing interests exist.*<br><br>**Authors with competing interests**<br><br>Enter competing interest details beginning with this statement:<br><br>*I have read the journal's policy and the authors of this manuscript have the following competing interests: [insert competing interests here]*<br><br>* typeset	
**Ethics Statement**<br><br>Enter an ethics statement for this submission. This statement is required if the study involved:<br><br>• Human participants<br>• Human specimens or tissue<br>• Vertebrate animals or cephalopods<br>• Vertebrate embryos or tissues<br>• Field research<br><br>Write "N/A" if the submission does not require an ethics statement.<br><br>General guidance is provided below. Consult the submission guidelines for detailed instructions. **Make sure that all information entered here is included in the Methods section of the manuscript.**	N/A

**Format for specific study types**<br><br>**Human Subject Research (involving human participants and/or tissue)**<br>• Give the name of the institutional review board or ethics committee that approved the study<br>• Include the approval number and/or a statement indicating approval of this research<br>• Indicate the form of consent obtained (written/oral) or the reason that consent was not obtained (e.g. the data were analyzed anonymously)<br><br>**Animal Research (involving vertebrate animals, embryos or tissues)**<br>• Provide the name of the Institutional Animal Care and Use Committee (IACUC) or other relevant ethics board that reviewed the study protocol, and indicate whether they approved this research or granted a formal waiver of ethical approval<br>• Include an approval number if one was obtained<br>• If the study involved *non-human primates*, add *additional details* about animal welfare and steps taken to ameliorate suffering<br>• If anesthesia, euthanasia, or any kind of animal sacrifice is part of the study, include briefly which substances and/or methods were applied<br><br>**Field Research**<br><br>Include the following details if this study involves the collection of plant, animal, or other materials from a natural setting:<br>• Field permit number<br>• Name of the institution or relevant body that granted permission	
**Data Availability**<br><br>Authors are required to make all data underlying the findings described fully available, without restriction, and from the time of publication. PLOS allows rare exceptions to address legal and ethical concerns. See the PLOS Data Policy and FAQ for detailed information.	Yes - all data are fully available without restriction

A Data Availability Statement describing where the data can be found is required at submission. Your answers to this question constitute the Data Availability Statement and **will be published in the article**, if accepted.<br><br>**Important:** Stating 'data available on request from the author' is not sufficient. If your data are only available upon request, select 'No' for the first question and explain your exceptional situation in the text box.<br><br>Do the authors confirm that all data underlying the findings described in their manuscript are fully available without restriction?	
**Describe where the data may be found in full sentences. If you are copying our sample text, replace any instances of XXX with the appropriate details.**<br><br>• If the data are **held or will be held in a public repository**, include URLs, accession numbers or DOIs. If this information will only be available after acceptance, indicate this by ticking the box below. For example: *All XXX files are available from the XXX database (accession number(s) XXX, XXX.).*<br>• If the data are all contained **within the manuscript and/or Supporting Information files**, enter the following: *All relevant data are within the manuscript and its Supporting Information files.*<br>• If neither of these applies but you are able to provide **details of access elsewhere**, with or without limitations, please do so. For example:<br><br>*Data cannot be shared publicly because of [XXX]. Data are available from the XXX Institutional Data Access / Ethics Committee (contact via XXX) for researchers who meet the criteria for access to confidential data.*<br><br>*The data underlying the results presented in the study are available from (include the name of the third party*	All relevant data are within the manuscript and its Supporting Information files

and contact information or URL).<br>• This text is appropriate if the data are owned by a third party and authors do not have permission to share the data.<br><br>* typeset	
Additional data availability information:	

Powered by Editorial Manager® and ProduXion Manager® from Aries Systems Corporation

Manuscript

Click here to access/download;Manuscript;Human error in SRs.docx

Error Rates of Human Reviewers During Abstract Screening in Systematic Reviews

Background

Automated approaches to improve the efficiency of systematic reviews are greatly needed. When testing any of these approaches, the criterion standard of comparison (gold standard) is usually human reviewers. Yet; human reviewers make errors in inclusion and exclusion of references.

Objectives

To determine citation false inclusion and false exclusion rates during abstract screening by pairs of independent reviewers. These rates can help in designing, testing and implementing automated approaches.

Methods

We identified all systematic reviews conducted between 2010 and 2017 by an evidence-based practice center in the United States. Eligible reviews had to follow standard systematic review procedures with dual independent screening of abstracts and full texts in which citation inclusion by one reviewer prompted automatic inclusion through the next level of screening. Disagreements between reviewers were reconciled via consensus or arbitration by a third reviewer. Data were extracted from web-based commercial systematic review software. A false inclusion or exclusion was defined as a decision made by a single reviewer that was inconsistent with the final included list of studies that underwent full text screening and consensus, data extraction and analysis.

Results

We analyzed a total of 139,467 citations that underwent 329,332 inclusion and exclusion decisions from 86 unique reviewers. The final systematic reviews included 5.48% of the references identified through bibliographic database search (95% confidence interval (CI): 2.38% to 8.58%). After abstract screening, the total error rate (false inclusion and false exclusion) was 10.76% (95% CI: 7.43% to 14.09%).

Conclusions

This study of 329,332 screening decisions made by a diverse group of systematic reviewers suggests important false inclusion and exclusion rates. When deciding about the validity of a future automated study selection algorithm, it is important to keep in mind that the gold standard is not perfect and that achieving error rates similar to humans is likely adequate and can save resources and time. A dual process of an automated tool and a human reviewer is recommended.

Background

Systematic review is a process to identify, select, synthesize and appraise all empirical evidence that fits pre-specified criteria to answer a specific research question. Since Archie Cochrane criticized lack of reliable evidence in medical care and called for “critical summary, by specialty or subspecialty, adapted periodically, of all relevant randomized control trials” in 1970s (1), systematic review has become the foundation of modern evidence based medicine. It is estimated that the annual publications of systematic reviews increased 2,728% from 1,024 in 1991 to 28,959 in 2014.(2)

Despite of the surging number of published systematic reviews in recent years, many systematic reviews employ suboptimal methodological approaches.(2-4) Rigorous systematic reviews require strict procedures with at least eight time-consuming steps.(5, 6) Significant time and resources are needed, with estimated 0.9 minutes, 7 minutes and 53 minutes spent per reference per reviewer on abstract screening, full text screening, and data extraction; respectively.(7, 8) 1,000 potential studies retrieved from literature search required 952 hours to complete.(9)Therefore, methods to improve efficiency of systematic reviews without jeopardizing the validity are greatly needed.

In recent year, innovations have been proposed to accelerate the process of systematic reviews, including methods to simplify steps of systematic reviews (e.g., rapid systematic reviews)(10-13), and technology to facilitate literature retrieval, screening, and extraction(7, 8, 14-20). Automation tools for systematic reviews, based on machine learning, text mining, and natural language processing, have particularly been popular with an estimated workload reduction from 30% to 70%.(14) Till July 2019, 39 tools have been completed and are available for “real-world” use.(21) However, assessment of these tools is ongoing.(19, 22) No large scale test has been conducted. No conclusions have been made on whether and how to implement these automation tools. Theoretically, assessment of the automation tools can be treated as a classification problem: to determine whether a citation should be included or excluded. The standard outcome metrics are used, such as sensitivity, specificity, area under curve (AUC), positive predictive value. The standard of comparison (a.k.a., gold standard) is usually human reviewers. Yet; human reviewers make errors. There is lack of evidence of human errors in the process of systematic reviews.

Thus, we conducted this study to determine citation selection error rate (false inclusion and false exclusion rates) in systematic reviews conducted by pairs of independent human reviewers during abstract screening. These rates are currently unknown and can help in designing, testing and implementing automated approaches.

Methods

Study design and data source

We searched all systematic reviews conducted by Mayo Clinic Evidence-based Practice Center in the United States. Eligible systematic reviews had to 1) be started and finished between June 1, 2010 and Dec 31, 2017; 2) follow standard systematic review procedures(5): dual independent screening of abstracts and full texts, citation inclusion by one reviewer prompted automatic inclusion through the next level of screening, disagreements between reviewers reconciled via consensus or arbitration by a third reviewer; 3) use a web-based commercial systematic review software (DistillerSR, Evidence Partners Incorporated, Ottawa, Canada) to facilitate abstracts and full texts screening; and 4) be led by at least one of the core investigators of the evidence-based practice center. The evidence-based practice center is one of the 12 evidence-based practice centers designated and funded by U.S. Agency for Healthcare Research and Quality (AHRQ). It specializes in conducting systematic reviews, meta-analysis, clinical practice guidelines, evidence dissemination and implementation, and related methodological research. The investigation team consists of a core group (10-15 investigators at any time period) and external collaborators with either methodological or content expertise. DistillerSR stores all decisions by reviewers including abstract screening, full text screening, and data extraction. This study is exempted by Mayo Clinic Institutional Review Board.

Outcomes

The main outcome of interest was error rate of human reviewers during abstract screening. An error was defined as a decision made by a single reviewer in abstract screening that was inconsistent (i.e., false inclusion or false exclusion) with the final included list of studies that underwent data extraction and analysis (see figure). We calculated error rate as the number of errors divided by the total number of screened abstracts (the total number of citations*2). We also estimated the overall abstract inclusion rate (defined as the number of eligible study after abstract screening divided by the total number of citations), and the final inclusion rate (defined as the number of the final included studies divided by the total number of citations).

Statistical analysis

We calculated the outcomes of interest for each eligible systematic review. The mean of the outcomes were the average outcomes of each study weighted by the inverse proportion to the variance of the denominator (total number of screened abstracts or total number of citations). All analyses were implemented with Stata version 15.1 (StataCorp LP, College Station, TX, USA).

Results

A total of 25 systematic reviews conducted between June 2010 and December 2017 were included in the analyses. These systematic reviews included 139,467 citations, representing 329,332 inclusion and exclusion decisions from 85 unique reviewers. 28 reviewers were core investigators from the evidence-based practice center; 57 were external collaborators with content or methodological expertise. Table 1 listed the characteristics of the included systematic reviews. Appendix S1 Table A listed the included systematic reviews.

Table 1. Characteristics of the included systematic reviews

Characteristics	N
Systematic reviews	25
Time period	June 2010 to December 2017
Databases included	At least Embase, Medline, and Cochrane Database of Systematic Review
Citations from literature search	139,467
Inclusion and exclusion decisions	329,332
decisions after abstract screening	278,934
decisions after full text screening	50,398
Systematic reviewers	85
From the core team	28
External content or methodological experts	57
Clinical area	
Cardiovascular medicine	1
Mental health	2
Primary care	3
Pulmonology and critical care	2
Cardiovascular medicine	1
Endocrinology	7
Hematology	2
Health care delivery research	4
Urology	3
Review question type	
Methodology	4
Diagnostic/Screening/Prognostic	4
Treatment	17

Abstract screening inclusion rate was 18.07% (95% CI: 12.65% to 23.48%) of the citations identified through literature search. Final inclusion rate after full text screening was 5.48% of the citations identified through literature search were included in the systematic review (95% confidence interval (CI):

2.38% to 8.58%).The total error rate was 10.76% (95% CI: 7.43% to 14.09%).The error rates and inclusion rates varied by clinical area and taxonomy of clinical questions (Table 2).

Table 2: Error and inclusion rates by topic area and type of review questions

	Error rate (95% CI)	Final inclusion rate (95% CI) dual process	Abstract inclusion rate (95% CI) dual process
Overall (n=25)	10.76% (7.43% to 14.09%)	5.48% (2.38% to 8.58%)	18.07% (12.65% to 23.48%)
Clinical Area			
Cardiovascular medicine (n=1)	17.73%	1.59%	23.92%
Mental health (n=2)	6.43% (0% to 18.19%)	2.10% (0% to 20.15%)	9.92% (0.77% to19.06%)
Primary care (n=3)	21.11% (5.15% to 37.08%)	5.39% (0% to 13.19%)	28.00% (20.85%, 35.15%)
Pulmonology and critical care (n=2)	6.68% (0% to 42.04%)	1.12% (0% to 2.43%)	9.13% (05 to 38.18%)
Cardiovascular medicine (n=1)	19.16%	1.93%	18.56%
Endocrinology (n=7)	12.23% (4.76% to 19.70%)	5.91% (2.89% to 8.94%)	20.40% (9.70% to 31.09%)
Hematology (n=2)	5.76% (0% to 24.27%)	6.69% (0% to 37.32%)	11.00% (0% to 40.13%)
Health care delivery research (n=4)	8.73% (0% to 29.03%)	2.18% (0% to 6.86%)	14.55% (0% to 43.35%)
Urology (n=3)	17.17% (0% to 36.07%)	23.77% (0% to 57.85%)	42.85% (24.92% to 60.79%)
Review question type			
Methodology	8.73% (0.00% to 29.03%	2.18% (0.00% to 6.86%)	14.55% (0% to 43.35%)
Diagnostic/Screening/Prognostic	14.97% (0% to 34.38%)	7.83% (3.56% to 12.11%)	25.99% (0% to 57.82%)
Treatment	10.57% (7.32% to 13.83%)	5.86% (1.47% to 10.26%)	17.64% (12.00% to 23.29%)

Discussion

In this cohort of 25 systematic reviews, covering 9 clinical areas and 3 types of clinical questions, a total of 329,332 screening decisions (inclusion vs. exclusion) were made by 85 human reviewers. The error rate (false inclusion and false exclusion) during abstract screening was 10.76%, which varied from 5.76% to 21.11%, depending on clinical areas and question types.

Implications

A rigorous systematic review follows strict approaches and requires significant resource and time to complete, which typically lasts 6-18 months by a team of human reviewers. Automation tools have the potential to mimic human activities in systematic review tasks and gained popularity in academia and industry. However, validity of the automation tools has yet to be established. It is intuitive to assume that these tools should achieve a zero error rate in order to be implemented to generate evidence used for decision-making (i.e., 100% sensitivity and 100% specificity). Yet, this target is unlikely achievable.

Human reviewers have been used as the “gold standard” in evaluating the automation tools. However, similar to those “gold standards” used in clinical medicine, no 100% accuracy can be achieved in reality. We found 10.76% error rate made by human reviewers in abstract screening (an error about 1 in 9 abstracts). This error rate also varied from topics and types of questions. Thus, when developing and refining an automation tool, achieving error rates similar to humans may be adequate. If this is the case, then these tools can serve as a single reviewer that gets paired with a second human reviewer.

Limitations

The sample size is relatively small, especially as we further stratify by clinical areas. The findings may not be generalizable to other systematic review questions or topics. The human reviewers who conducted these systematic reviews had a wide range of content knowledge and methodological experience (from minimum to over 10 years), which can be quite different from other review teams. In our practice, citations from abstract screening were automatically included when conflicts between two independent reviewers emerged. The findings resulting from this approach can be different from those of the teams who resolve conflicts in abstract screening. When both reviewers agree on excluding an abstract, this abstract disappears from the process; thus, a dual erroneous exclusion cannot be assessed. Lastly, while we call judgments in study selection that are inconsistent with the final inclusion as errors, we acknowledge that these errors could be due to poor reporting and insufficient data provided in the published abstract. Thus, they may not be avoidable and they are not the fault of the reviewer.

Conclusions

This study of 329,332 abstract screening decisions made by a large, diverse group of systematic reviewers suggests important false inclusion and exclusion rates. When deciding about the validity of a future automated study selection algorithm, it is important to keep in mind that the gold standard is not perfect and that achieving error rates similar to humans is likely adequate and can save resources and time. A dual process of an automated tool and a human reviewer is recommended.

Reference

1. Cochrane AL. 1931-1971: a critical review, with particular reference to the medical profession. Medicines for the year. 2000;1979:1.
2. Ioannidis JP. The Mass Production of Redundant, Misleading, and Conflicted Systematic Reviews and Meta-analyses. Milbank Q. 2016;94(3):485-514.
3. Page MJ, Altman DG, McKenzie JE, Shamseer L, Ahmadzai N, Wolfe D, et al. Flaws in the application and interpretation of statistical analyses in systematic reviews of therapeutic interventions were common: a cross-sectional analysis. J Clin Epidemiol. 2018;95:7-18.
4. Baudard M, Yavchitz A, Ravaud P, Perrodeau E, Boutron I. Impact of searching clinical trial registries in systematic reviews of pharmaceutical treatments: methodological systematic review and reanalysis of meta-analyses. BMJ. 2017;356:j448.
5. Higgins JP, Green S. Cochrane handbook for systematic reviews of interventions: John Wiley & Sons; 2011.
6. Murad MH, Montori VM, Ioannidis JP, Jaeschke R, Devereaux P, Prasad K, et al. How to read a systematic review and meta-analysis and apply the results to patient care: users' guides to the medical literature. Jama. 2014;312(2):171-9.
7. Wang Z, Asi N, Elraiyah TA, Abu Dabrh AM, Undavalli C, Glasziou P, et al. Dual computer monitors to increase efficiency of conducting systematic reviews. J Clin Epidemiol. 2014;67(12):1353-7.
8. Wang Z, Noor A, Elraiyah T, Murad M, editors. Dual monitors to increase efficiency of conducting systematic reviews. 21st Cochrane Colloquium; 2013.
9. Allen IE, Olkin I. Estimating time to conduct a meta-analysis from number of citations retrieved. JAMA. 1999;282(7):634-5.
10. Khangura S, Konnyu K, Cushman R, Grimshaw J, Moher D. Evidence summaries: the evolution of a rapid review approach. Syst Rev. 2012;1:10.
11. Hailey D, Corabian P, Harstall C, Schneider W. The use and impact of rapid health technology assessments. International journal of technology assessment in health care. 2000;16(2):651-6.
12. Patnode CD, Eder ML, Walsh ES, Viswanathan M, Lin JS. The use of rapid review methods for the US Preventive Services Task Force. American journal of preventive medicine. 2018;54(1):S19-S25.
13. Ganann R, Ciliska D, Thomas H. Expediting systematic reviews: methods and implications of rapid reviews. Implement Sci. 2010;5:56.
14. O'Mara-Eves A, Thomas J, McNaught J, Miwa M, Ananiadou S. Using text mining for study identification in systematic reviews: a systematic review of current approaches. Systematic reviews. 2015;4(1):5.
15. Li D, Wang Z, Wang L, Sohn S, Shen F, Murad MH, et al. A Text-Mining Framework for Supporting Systematic Reviews. Am J Inf Manag. 2016;1(1):1-9.
16. Li D, Wang Z, Shen F, Murad MH, Liu H, editors. Towards a multi-level framework for supporting systematic review—A pilot study. 2014 IEEE International Conference on Bioinformatics and Biomedicine (BIBM); 2014: IEEE.
17. Alsawas M, Alahdab F, Asi N, Li DC, Wang Z, Murad MH. Natural language processing: use in EBM and a guide for appraisal. BMJ Evidence-Based Medicine. 2016;21(4):136-8.
18. Cohen AM, Hersh WR, Peterson K, Yen P-Y. Reducing workload in systematic review preparation using automated citation classification. Journal of the American Medical Informatics Association. 2006;13(2):206-19.

19. Beller E, Clark J, Tsafnat G, Adams C, Diehl H, Lund H, et al. Making progress with the automation of systematic reviews: principles of the International Collaboration for the Automation of Systematic Reviews (ICASR). Syst Rev. 2018;7(1):77.
20. O'Connor AM, Tsafnat G, Gilbert SB, Thayer KA, Shemilt I, Thomas J, et al. Still moving toward automation of the systematic review process: a summary of discussions at the third meeting of the International Collaboration for Automation of Systematic Reviews (ICASR). Syst Rev. 2019;8(1):57.
21. SR Toolbox 2019 [Available from: http://systematicreviewtools.com/index.php.
22. O'Connor AM, Tsafnat G, Thomas J, Glasziou P, Gilbert SB, Hutton B. A question of trust: can we build an evidence base to gain trust in systematic review automation technologies? Systematic Reviews. 2019;8(1):143.

Click here to access/download

Supporting Information

PLoS One Supporting Information Appendix S1.docx
