# Supplementary material for: Error rates of human reviewers during abstract screening in systematic reviews
Source: PLoS One. 2020 Jan 14;15(1):e0227742. doi: 10.1371/journal.pone.0227742 (PMC6959565; doi:10.1371/journal.pone.0227742)
Supplement: Attachment — Submitted filename: PONE-D-19-26633_comments.pdf [file pone.0227742.s003.pdf]

PONE-D-19-26633_reviewer

Error Rates of Human Reviewers During Abstract Screening in Systematic Reviews

By: Zhen Wang et al.

General comments:

Material and Methods section, study selection:

It is not clearly stated by the authors whether they did or not a comparison between selection by a software (DistillerSR) and a human team, the latter considered as the gold standard for study screening.

Material and Methods section, outcomes:

Did you check each systematic review included in the citations of your study to decide whether the studies included and excluded were the right ones or not? Is that what you mean?

I would like to see a more detailed definition on the method you used to decide whether a specific study should be included or excluded. It is not clear at all how you made this final decision on a particular study. Let's suppose that a study was excluded from the data extraction, how you can find that study was a false exclusion? This is what I mean when asking for more details to have clarity on your methodology.

Figure is missing.

Material and Methods section, statistical analysis:

It is not clear how the variance for weighting the outcome average was calculated.

The description of statistical methods is not clear.

Is there any potential bias selection given that you only included studies that used DistillerSR for study screening?

Conclusion:

The final conclusive sentence is not supported by the data. It is necessary to clarify whether authors did a comparison between automated and human revision methods to decide inclusion/exclusion of studies during a systematic review process.

Please, check notes on the revised manuscript.
