# Supplementary material for: Error rates of human reviewers during abstract screening in systematic reviews
Source: PLoS One. 2020 Jan 14;15(1):e0227742. doi: 10.1371/journal.pone.0227742 (PMC6959565; doi:10.1371/journal.pone.0227742)
Supplement: Attachment — Submitted filename: Response to Reviewers.docx [file pone.0227742.s004.docx]

Dear Dr. Bencharit,

Thank you for the review of our manuscript. We have attached our point by point response. Please let us know if you have additional suggestions/comments.

Best,

Zhen Wang, Ph.D.

Associate Professor of Health Services Research, Mayo Clinic College of Medicine and Science

Associate Director, Mayo Clinic Evidence-based Practice Center

Robert D. and Patricia E. Kern Center for the Science of Health Care Delivery

Division of Health Care Policy and Research, Department of Health Sciences Research

Phone: 507-538-6153

Fax: 507-284-1731

Email: wang.zhen@mayo.edu

Reviewers’ comments

General comments:

Material and Methods section, study selection:

It is not clearly stated by the authors whether they did or not a comparison between selection by a software (DistillerSR) and a human team, the latter considered as the gold standard for study screening.

**Response:** We did not compare the performance between human reviewers and the software. As stated, the objective for this study was to determine citation false inclusion and false exclusion rates during abstract screening by pairs of independent reviewers. We clarified this point in the Methods section.

Material and Methods section, outcomes:

Did you check each systematic review included in the citations of your study to decide whether the studies included and excluded were the right ones or not? Is that what you mean?

**Response:** Yes, this is correct. The included or excluded studies after abstract screening were compared to the final set of the included studies (aka. the “right” ones). In this revision, we also attached the figure. Hopefully, the figure can clarify this issue.

I would like to see a more detailed definition on the method you used to decide whether a specific study should be included or excluded. It is not clear at all how you made this final decision on a particular study. Let’s suppose that a study was excluded from the data extraction, how you can find that study was a false exclusion? This is what I mean when asking for more details to have clarity on your methodology.

**Response:**  We followed the standard systematic reviews procedures: 1) dual independent screening of abstracts and titles, abstract inclusion by one reviewer prompted automatic inclusion for full text screening; 2) dual independent screening of full text, disagreements between reviewers reconciled via consensus or arbitration by a third reviewer. The final included list of studies consisted of the studies after abstract screening, and full texting screening.

In data extraction, we did not exclude studies. The studies were determined after full text screening. An error was defined as a decision made by a single reviewer in abstract screening that was inconsistent (i.e., false inclusion or false exclusion) with the final included list of studies that underwent abstract screening, and full texting screening and were eligible for data extraction and analysis (see figure).

We clarified these points in the revision.

Figure is missing.

**Response:** We added the figure.

Material and Methods section, statistical analysis:

It is not clear how the variance for weighting the outcome average was calculated.

The description of statistical methods is not clear.

**Response:** we used the following formula for the variance.

$$V=\left\{ \frac{n}{\left[ sum w_{i}\left( n - 1 \right) \right]} \right\}sum w_{i} \left( x_{i}- xbar \right)^{2}$$

$xbar$ =weight mean

We add this in the methods.

Is there any potential bias selection given that you only included studies that used DistillerSR for study screening?

**Response:** DistillerSR is a web-based systematic review software that facilitates systematic review procedures (e.g. abstracts and full texts screening). The literature search, study screening, data extraction and other steps in systematic reviews are done by human reviewers. distillerSR did not conduct any automation in the included systematic reviews. We don’t see any potential bias due to DistillerSR. We clarified this point in the revision.

Conclusion:

The final conclusive sentence is not supported by the data. It is necessary to clarify whether authors did a comparison between automated and human revision methods to decide inclusion/exclusion of studies during a systematic review process.

**Response:** We did not compare the performance between automated systematic review methods and human reviewers. Instead, we evaluated the error rates made by human reviewers (aka, the gold standard). Our main conclusion was that the gold standard is not perfect and that achieving error rates similar to humans is adequate for a future automated algorithm. We clarified this point in the revision.

Please, check notes on the revised manuscript.

**Response:** We listed our responses here.

1. Do not start a paragraph with number, use text. For instance: One thousand potential studies…

**Response:** We corrected all of these.

2. Please, check that there are some citations after the period to finish the sentence, and some other before the period.

**Response:** We changed so all citations are after the period.

3. When you state "automation" for abstract selection or final selection, you mean that this software was used to select the studies to be included or excluded? Was there a comparison against the "human" gold standard for selection?

**Response:** DistillerSR is a web-based systematic review software that hosts data and facilitates systematic review procedures (e.g. abstracts and full texts screening). The literature search, study screening, data extraction and other steps in systematic reviews are done by human reviewers. distillerSR did not conduct any automation in the included systematic reviews. We clarified in the revision.

4. Please, move this sentence describing the Center to the second line of the paragraph, just after " … United States." That is because you are describing here the Center and its team.

**Response:** We moved this sentence as suggested.

5. I could not find the figure. On the other hand, what is the figure number?

**Response:** We added Figure 1 in the submission.

6. Would it be possible to give more details on how you calculated the variance of denominator? Is this the variance for the average number of total screened abstracts or total number of citations of all studies? This variance calculation is not clear how was obtained.

**Response:** We used the following formula for the variance.

$$V=\left\{ \frac{n}{\left[ sum w_{i}\left( n - 1 \right) \right]} \right\}sum w_{i} \left( x_{i}- xbar \right)^{2}$$

$xbar$ =weight mean

We add this in the methods.

7. It is clear the criteria you used to select the studies. However, do you have any comments on the low number of studies? It is almost 8 years, so I would think that the number of published SR could be more than this.

**Response:** Not all of our systematic reviews were implemented in DistillerSR. We excluded those systematic reviews in this study.

8. Table 1. What does N mean?

**Response:** It should be “results”. We corrected this.

9. Table 2. Following the text, the order of the columns should be: Final inclusion rate, Abstract inclusion rate, Error rate

**Response:** We changed the order of the columns as suggested.

10. Are there any citation in the discussion section to support your ideas?

**Response:** We added citations as suggested.

11. This conclusion is just what is not supported from your data, as it was not clear whether you did a comparison between the inclusión/exclusion using DistillerSR and a human decision.

**Response:** We agree and deleted this sentence.

Reviewer #1: Thank you for this study which contributes to our knowledge of the process of conducting systematic reviews.

Data Availability: The list of systematic reviews examined is provided; however, there are no data regarding the full sets of references obtained from the original searches of the examined systematic reviews, nor the inclusion/exclusion decisions of each reviewer for each systematic review. Therefore, readers would not be able to replicate this analysis with the data currently provided.

**Response:** We now included the de-identified dataset in the revision.

Statistical Analysis: the descriptive statistics were calculated correctly.

**Response:** Thank you.

Background: You mention that systematic reviews employ suboptimal methodological approaches and the potential for human errors; however, you do not acknowledge that automated systems and algorithms could introduce errors, potentially systematic errors which could introduce bias into a systematic review, such as that found in this study <https://science.sciencemag.org/content/366/6464/447>

**Response:** We thank the reviewer for this great suggestion. We added the point to the introduction.

Methods: Consider explaining/justifying the eligibility criteria #3 use a single software program, Distiller SR - were reviews excluded because they used a different software program? For eligibility criteria #2 - was the citation inclusion by one reviewer prompting automatic inclusion happen at only the abstract screening level (vs. full text screening)? under what circumstances were disagreements between reviewers reconciled via consensus or arbitration.

**Response:** DistillerSR was used to facilitate abstracts and full texts screening and track all inclusion and exclusion decisions made by human reviewers. So for other systematic reviews, we, no longer, have relevant data for the study.

For criteria 2, we did not resolve conflicts in abstract screening. The conflicts were automatically included for full text screening. In full text screening, we resolved the conflicts through consensus between reviewers or arbitration by a third reviewer. We clarified these points in the revision.

Results: Consider breaking out the error rate to errors of exclusion and errors of inclusion, as these may be different and of interest to readers and would allow you to provide rates of specificity and sensitivity, typical metrics for classification problems as you mention in the Background section and as was done in the study by Bannach-Brown referenced below.

Further, I would like to see a breakdown of errors from the abstract screening vs. the full text screening (and within this stratification, report exclusion vs. inclusion errors). This is important and interesting, because as you note in the limitations, the errors of inclusion at the abstract screening level reflect the fact that more information is needed to make a decision, rather than commission of an error by the reviewer.

**Response:** These are excellent points. However, with our data, we are not able to provide additional results as suggested. First, false exclusion is biased as “When both reviewers agree on excluding an abstract, this abstract disappears from the process; thus, a dual erroneous exclusion cannot be assessed.” (limitation, line 152 and Fig 1). We also did not track or store the conflicts after full text screening. We don’t know the error rate from full text screening. We added this point as a limitation.

Limitations: You note that "In our practice, citations from abstract screening were automatically included when conflicts between two independent reviewers emerged" - perhaps note that this may have resulted in a spuriously lower error rate than "truth."

**Response:** We don’t believe this practice led to “spuriously lower error rate”. The more common practice is to resolve the conflicts during abstract screening. The errors made by reviewers remain stable, not higher or lower. However, our approach may have led to higher e abstract and final inclusion rates. We clarified this in the revision.

Consider referencing the following article in the background or implications as this study calculated error rates from a Machine Learning screening algorithm: Bannach-Brown, A., Przybyła, P., Thomas, J., Rice, A. S., Ananiadou, S., Liao, J., & Macleod, M. R. (2019). Machine learning algorithms for systematic review: reducing workload in a preclinical review of animal studies and reducing human screening error. Systematic reviews, 8(1), 23.

**Response:** We thank the reviewer for bringing this study to our attention. We cited this study in the revision.

Limitations or perhaps conclusions: As this is a novel study, consider contextualizing the findings as an initial calculation of human errors observed in a small number of systematic reviews and discussing the need for replication of this study using reviews conducted by other EPCs or other non-EPC institutions, using different software, and most important to increase the sample size of studies used to inform our knowledge of valid human error rates.

**Response:** We agree and added this point in the limitation.

Typo: 3rd paragraph of background section, In recent year should be In recent years

**Response:** We corrected this.

Reviewer #2: Dear authors - Thank you for investigating this question. It's of interest to me.

I agree that the sample is small and I wondered how meaningful the 10% error rate is. It's expected that human reviewers will make mistakes and if each reviewer errors on 10% of decisions, then... what? That's one reason why there are two reviewers. However, the use of that number as a benchmark to test automated systems puts it in an interesting context. If the automated process is as effective as a human reviewer, then we can save significant human time by eliminating one of the reviewers. But, again, the sample is small, and I imagine the type of question can influence the error rate, and some screening questions might be more conducive to human review / automation. In other words, a 10% error rate for one question may vary drastically for another. Some of this is touched on in the 'implications' section, which I'd like to see expanded, but I understand that such discussion can easily go beyond the study.

Overall, the small sample and the topic variability in the sample and the variability of the screening questions in each of the reviews in the sample lead me to question the significance of the 10%. And that makes me question its utility as a benchmark. But it's a thought-provoking topic and the authors use an interesting method to address it (Distiller data).

Thank you, by the way, for including the complete list of systematic reviews included in the analysis. This helps with reproducibility.

**Response:** We thank the reviewer for this important issue. This study evaluated 139,467 citations, representing 329,332 inclusion and exclusion decisions from 85 unique reviewers. We agree that the number of systematic reviews is small, especially as we further stratify by clinical areas/type of review questions. We added this as a limitation and emphasized that this study is a first step to evaluate human errors in systematic reviews. Future studies need to evaluate different systematic review approaches (e.g., rapid systematic review, scoping review), clinical areas, and review questions. It is also important to increase the number of systematic reviews involved in the evaluation and include other EPC or non-EPC institutions.

Reviewer #3: Dear Authors,
I have attached a revised version of your manuscript and a PDF file including my specific comments. My major concerns are related to the methdology you followed in your study.
Please, check both.
Best regards.

**Response:** Our responses are listed at the beginning of the file.
